# Enhanced cytotoxicity in triple-negative and estrogen receptor-positive breast adenocarcinoma cells due to inhibition of the transient receptor potential melastatin-2 channel

**DOI:** 10.3892/or.2015.4131

**Published:** 2015-07-14

**Authors:** DAVID W. KOH, DANIEL P. POWELL, STEVEN D. BLAKE, JOY L. HOFFMAN, MANDI M. HOPKINS, XIAOXING FENG

**Affiliations:** 1 Department of Pharmaceutical and Biomedical Sciences, Ohio Northern University, Ada, OH 45810, USA; 2 Department of Pharmaceutical Sciences, Washington State University, Pullman, WA 99164, USA

**Keywords:** triple-negative breast cancer, estrogen receptor-positive breast cancer, transient receptor potential, TRPM2, DNA damage, cell death, chemotherapy

## Abstract

We previously demonstrated a unique protective role for the transient receptor potential, melastatin-2 (TRPM2) cation channel in breast cancer cells. In the present study, we investigated the chemotherapeutic effects elicited by inhibiting this protective role in metastatic breast adenocarcinoma cells. TRPM2 inhibition led to dose-dependent increases in MDA-MB-231 breast adenocarcinoma cell death after treatment with doxorubicin or the DNA-methylating agent, N-methyl-N'-nitro-N-nitrosoguanidine. Similar results were observed after RNAi silencing of TRPM2 in these cells after doxorubicin treatment. However, TRPM2 RNAi silencing also led to increased MCF-7 breast adenocarcinoma cell death after tamoxifen treatment, yet not in non-cancerous human mammary epithelial cells. These results thus revealed that TRPM2 inhibition selectively increased cytotoxicity in a triple-negative and an estrogen receptor-positive breast cancer cell line, with minimal deleterious effects in non-cancerous breast cells. Analysis of DNA damage revealed enhanced DNA damage levels in MCF-7 cells treated with doxorubicin due to TRPM2 inhibition. Analysis of cell death demonstrated that inhibition of apoptosis, caspase-independent cell death or autophagy failed to significantly reduce cell death induced by TRPM2 inhibition and chemotherapy. These results indicate that TRPM2 inhibition activates alternative pathways of cell death in breast cancer cells. Taken together, our results provide significant evidence that TRPM2 inhibition is a potential strategy to induce triple-negative and estrogen receptor-positive breast adenocarcinoma cell death via alternative cell death pathways. This is expected to provide a basis for inhibiting TRPM2 for the improved treatment of breast cancer, which potentially includes treating breast tumors that are resistant to chemotherapy due to their evasion of apoptosis.

## Introduction

The treatment options for breast cancer are based in part upon the classification of the tumor molecular subtype. Of the three molecular subtypes of invasive breast cancer, triple-negative (TN) is the most aggressive and is associated with the worst prognosis for patients (1–3). Moreover, patients with this form of breast cancer are typically not candidates for hormonal therapy or human epidermal growth factor 2 (HER2)-targeted therapy (4). Another molecular classification is estrogen receptor-positive (ER^+^) breast cancer. This molecular subtype of breast cancer is associated with greater treatment options than TN, such as treatment with the anti-estrogen, tamoxifen (5,6). Furthermore, patients diagnosed with ER^+^ breast cancer have an improved prognosis as compared to patients with TN breast cancer (7). This includes greater response rates to chemotherapeutic treatments, higher median survival rates, and increased lifespan (8,9). However, a complicating factor in the treatment of patients with ER^+^ breast cancer, as well as those with TN breast cancer, is the phenomenon that certain TN and ER^+^ breast tumors are resistant to chemotherapeutic treatments. While there are several mechanisms in which such breast tumors fail to respond to chemotherapeutic treatments, one in particular is the ability to evade the apoptotic cell death induced by chemotherapeutic agents via inhibition of pro-apoptotic mediators or activation of anti-apoptotic proteins (10–12). Therefore, improved therapies for TN and ER^+^ breast cancers, and breast tumors resistant to current therapies, are paramount in order to successfully treat these devastating conditions and improve the prognosis for breast cancer patients in the future.

An emerging target for the improved treatment of breast cancer is the transient receptor potential, melastatin-2 (TRPM2) channel. TRPM2 is a non-specific cation channel activated by oxidative stress (13). Once activated, it gates sodium, potassium and calcium ions into the cell (14). Among these cations, calcium has been studied the most extensively in regards to cell death. Calcium influx due to TRPM2 activation has been shown to facilitate apoptosis and caspase-independent cell death pathways (15–17). Due to this positive regulation of cell death pathways by TRPM2 in noncancerous cells, TRPM2 is thus targeted for pharmacologic inhibition for the protective effects such agents provide in these cells (16,18,19). However, in cancer cells, TRPM2 appears to have a unique role. Two TRPM2 mRNA transcripts, one antisense transcript and one truncated TRPM2 transcript were shown to be increased in metastatic melanoma cell lines (20). TRPM2 isoforms were shown to be overexpressed in several cancers, including melanoma, breast, and lung cancer (20,21). In prostate and breast cancer cells, TRPM2 appears to play a protective role. Inhibition or RNAi silencing of TRPM2 in prostate cancer cells led to decreased proliferation, while equivalent treatments failed to decrease proliferation in noncancerous prostate cells (22). Our previous study demonstrated that TRPM2 inhibition or RNAi silencing caused decreased proliferation and increased levels of DNA damage in breast adenocarcinoma cells, yet no significant effects were noted in non-cancerous mammary epithelial cells (23). Furthermore, a recent study demonstrated that RNAi knockdown of TRPM2 led to decreased growth of human xenograft neuroblastoma tumors in athymic nude mice, which thus demonstrated that TRPM2 modulates tumor growth in neuroblastoma (21). Thus, there is increasing evidence which demonstrates that inhibition of TRPM2 provides chemotherapeutic effects in cancer cells, with little or no harmful effects in non-cancerous cells. TRPM2 therefore appears to be a unique target in multiple cancers, where its pharmacologic inhibition can potentially provide an innovative strategy to selectively eradicate the tumors associated with those types of cancers.

However, in breast cancer cells, a comprehensive understanding of the chemotherapeutic effects elicited by TRPM2 inhibition is not completely known. Furthermore, since TRPM2 exacerbates cell death in normal cells (24) but has a novel protective role in breast cancer cells (23), a complete understanding of the cell death mechanisms initiated after TRPM2 inhibition in breast cancer cells is not known. In the present study, we investigated the chemotherapeutic effects produced in breast adenocarcinoma cells via TRPM2 inhibition or RNAi silencing, with a particular focus on cell death pathways, DNA damage levels, and the ability of TRPM2 inhibition to enhance the cytotoxicity of currently used chemotherapeutic agents-of-choice in different molecular subtypes of breast cancer. The present study found that TRPM2 inhibition enhanced the cytotoxicity of chemotherapeutic agents currently utilized to treat TN and ER^+^ breast cancer. Also, TRPM2 inhibition induced alternative pathways of cell death in these breast cancer cells. Since these results have not been previously reported in breast cancer cells, we thus report important findings which further identify the targeting of TRPM2 as a potential strategy to improve the chemotherapeutic treatment of breast cancer patients.

## Materials and methods

### Chemicals

*N*-(*p*-amylcinnamoyl)anthranilic acid (ACA), maintained as a 50 mM stock solution in dimethylsulfoxide (DMSO), 2-aminoethoxydiphenyl borate (2-APB; 75 mM stock solution in DMSO), aristolochic acid (75 mM stock solution in DMSO) and 3-methyladenine (3-MA; 50 mM stock solution in DMSO) were purchased from Sigma (St. Louis, MO, USA). N-Methyl-N'-nitro-N-nitrosoguanidine (MNNG; 0.5 M stock solution in DMSO) was purchased from AccuStandard (New Haven, CT, USA). Doxorubicin hydrochloride and tamoxifen citrate were purchased from Thermo Scientific (Waltham, MA, USA). Q-VD-OPh was purchased from R&D Systems (Minneapolis, MN, USA) and maintained as a 50 mM stock solution in DMSO. Propidium iodide (PI) (10 mg/ml solution) was purchased from Thermo Scientific. ApoScreen Annexin V-fluorescein isothiocyanate (FITC) was purchased from Southern Biotech (Birmingham, AL, USA).

### Cell lines and cell culture reagents

MCF-10A (human mammary epithelial), MCF-7 (human breast adenocarci-noma), and MDA-MB-231 (human breast adenocarcinoma) cell lines were purchased from the American Type Culture Collection (ATCC; Manassas, VA, USA). The human mammary epithelial cell (HMEC) line was purchased from Lonza (Walkersville, MD, USA). Dulbecco's modified Eagle's medium (DMEM) and fetal bovine serum (FBS; US origin, certified) were purchased from HyClone (Logan, UT, USA). Mammary epithelial growth medium (MEGM), which is specialized growth medium for HMEC and MCF-10A cells, was purchased from Lonza. Trypsin-EDTA (0.05%/0.53 mM), penicillin-streptomycin solution (10,000 IU/ml penicillin, 10,000 *μ*g/ml streptomycin), and 200 mM L-glutamine solution were purchased from Corning (Manassas, VA, USA).

### Other reagents

Opti-MEM reduced serum medium and Lipofectamine 2000 reagent were purchased from Invitrogen (Carlsbad, CA, USA). Protease inhibitor cocktail tablets (Complete Mini EDTA-free) were purchased from Roche (Mannheim, Germany). Primary antibodies utilized were poly-clonal rabbit anti-human TRPM2 antibody (cat. #A300-414A; Bethyl Laboratories, Montgomery, TX, USA), polyclonal rabbit anti-human β-actin (cat. #600-401-886), polyclonal rabbit anti-human apoptosis-inducing factor (AIF) (cat. #200-401-985) (both from Rockland Immunochemicals, Limerick, PA, USA) and monoclonal mouse anti-human poly(ADP-ribose) glycohydrolase (PARG) clone D8B10 (cat. #MABS61; Millipore). Two secondary antibodies, horseradish peroxidase (HRP)-conjugated goat anti-rabbit and HRP-conjugated rabbit anti-mouse were purchased from Jackson ImmunoResearch (West Grove, PA, USA). The CometAssay kit, which includes alkaline lysis solution, LMAgarose, 2-well CometSlides and SYBR-Green was purchased from Trevigen (Gaithersburg, MD, USA).

### Cell culture

MDA-MB-231 and MCF-7 cells were grown and maintained in DMEM supplemented with 10% FBS and 2 mM L-glutamine. Non-cancerous human mammary cells (MCF-10A and HMEC) were cultured in MEGM specialty medium which contains mammary epithelial cell basal medium (MEBM), 5 *μ*g/ml bovine pituitary extract, 0.01 *μ*g/ml human epidermal growth factor, 0.5 *μ*g/ml hydrocortisone, GA-1000 (60 *μ*g/ml gentamicin and 0.03 *μ*g/ml amphotericin B) and 5 *μ*g/ml insulin. All cultures were incubated at 37°C in 5% CO_2_ until treatments and analyses. Every two days in culture, cells were washed once with phosphate-buffered saline pH 7.2 (PBS) and cultured in fresh growth medium.

### RNA interference

The silencing of TRPM2 by RNAi was performed as previously reported using small-interfering RNA (siRNA) specific to nucleotides 4574–4594 in human T R PM2 (5′-AUAGAUCAGGAACUCCGUCUC-3′) (22). This RNA oligo was purchased as duplexed RNA (Integrated DNA Technologies, Coralville, IA, USA), resuspended in RNase-free water at a final concentration of 40 *μ*M and stored at -20°C. The silencing of AIF by RNAi was performed as previously described using sense (5′-CUUGUUCCAGCGAUGGCAUtt-3′) and antisense (5′-AUGCCAUCGCUGGAACAAGtt-3′) RNA oligos that target nucleotides 151–171 in human AIF (25). The silencing of PARG by RNAi was performed as previously described using RNA oligos (5′-AAATGGGACTTTACAGCTTTG-3′) that target nucleotides 1960-1980 in human PARG (26). Oligos 5′-AGACAGAAGACAGAUAGGCtt-3′ (sense) and 5′-GCCUAUCUGUCUUCUGUCUtt-3′ (antisense) were used for all negative controls (25). Anti-AIF and anti-PARG RNA oligos were purchased as duplexed RNA from Sigma-Aldrich, resuspended in RNase-free H_2_O at a final concentration of 20 *μ*M and stored at -20°C.

For RNAi transfections, cells were plated in 24-well plates containing 0.5 ml medium/well without antibiotics. On the day of transfection, cells were ~50–70% confluent. For RNAi transfection, duplex siRNA was added to 50 *μ*l Opti-MEM medium and 1 *μ*l Lipofectamine 2000 was added to 50 *μ*l Opti-MEM. The two solutions were gently mixed and incubated at room temperature for 20 min. Final concentrations of siRNA were 100 nM for TRPM2, 20 nM for AIF and 40 nM for PARG. The mixtures were added dropwise to each well and cells were cultured for an additional 48 h.

### Whole cell lysate extraction

Transfected cells were harvested by trypsinization, centrifuged at 112 × g for 5 min at 4°C, and washed once with 0.2 ml ice-cold PBS. The pellet was resuspended in 0.2 ml lysis buffer containing 25 mM Tris-HCl (pH 7.5), 150 mM NaCl, 1 mM EDTA, 1 mM EGTA, 1% NP-40 and protease inhibitors (Complete Mini EDTA-free tablets). Suspensions were incubated for 30 min on ice and vortexed every 10 min. The cell lysates were cleared by centrifugation at 16,000 × g for 10 min. The protein concentration for all sample lysates was then obtained using the Pierce BCA protein assay kit (Thermo Scientific). After BCA quantification, sodium dodecyl sulfate-polyacrylamide gel electrophoresis (SDS-PAGE) sample buffer (50 mM Tris-HCl pH 6.8, 1% SDS, 2.5% glycerol, 0.005% bromophenol blue and 100 mM dithiothreitol) were added to the supernatants. Samples were then heated for 2 min at 95°C in a digital dry bath incubator (Labnet International, Edison, NJ, USA).

### Cell death assay

Thirty minutes after the cells were pretreated with TRPM2 inhibitors or cell death inhibitors, or 48 h after cells were transfected with siRNA, the cells were treated with doxorubicin, MNNG or tamoxifen at the indicated doses and exposures. Approximately 16–20 h after treatment, the cells were harvested, washed with PBS and resuspended in Annexin V binding buffer (10 mM HEPES, pH 7.4, 140 mM NaCl, 2.5 mM CaCl_2_) to ~1×10^6^ cells/ml. For each 100 *μ*l of cell suspension, 4 *μ*l of ApoScreen Annexin V-FITC-conjugated solution (Southern Biotech) and 1 *μ*l of 100 *μ*g/ml PI solution were added. The cells were then incubated at room temperature for 15 min and 400 *μ*l Annexin V binding buffer was added. Cell death was then measured by quantifying the percentage of cells that exhibited Annexin V-FITC and/or PI fluorescence using a flow cytometer (BD Accuri, Ann Arbor, MI, USA). Total cell death was quantified by adding the percentage of cells detected in the upper left (PI), upper right (PI + Annexin V-FITC), and lower right (Annexin V-FITC) quadrants in the FACS dot plots. For pretreatment with cell death inhibitors, the cells were treated with 5 mM 3-MA or 40 *μ*M Q-VD-OPh 30 min prior to chemotherapeutic treatments. Treatment with 3-MA or Q-VD-OPh continued during all treatments and until FACS analyses were performed.

### Immunoblotting

Approximately 40 *μ*g of total protein from each lysate was separated on a 7.5% SDS-PAGE gel. The proteins were transferred to 0.45 *μ*m nitrocellulose (Protran BA85 85; GE Healthcare, Pittsburgh, PA, USA) by semi-dry transfer at 25 V for 1 h using a Trans-blot SD apparatus (Bio-Rad, Hercules, CA, USA). Membranes were blocked with PBS containing 0.05% Tween-20 (PBST) and 5% milk at room temperature for 1 h, and then incubated with primary antibodies (1:1,000 anti-TRPM2, 1:2,000 β-actin, 1:2,000 anti-AIF, or 1:2,000 anti-PARG) in PBST + 5% milk overnight at 4°C with shaking. Membranes were then washed with PBST three times and incubated with HRP-conjugated goat anti-rabbit (1:5,000) or HRP-conjugated rabbit anti-mouse antibody (1:5,000) for 1 h. The membranes were washed as before and chemiluminescence was initiated using the SuperSignal West Pico Chemiluminescent Substrate or SuperSignal West Femto Chemiluminescent Substrate (Thermo Fisher Pierce). Chemiluminescent detection was then accomplished on a ChemiDoc XRS gel imaging system (Bio-Rad).

### Comet assays

Comet assays were performed as previously described using the CometAssay ES system and the manufacturer's protocol for alkaline electrophoresis (27). Briefly, MCF-7 cells in 24-well tissue culture plates were incubated overnight in 0.5 ml growth medium, and then treated the next day with 2 *μ*M doxorubicin with or without a 30-min pretreatment with 100 *μ*M 2-APB. After collection by trypsinization 24 h later, a cell concentration of 1×10^5^ cells/ml was combined with low melting point agarose at a ratio of 1:10 (v/v). Approximately 50 *μ*l of the agarose/cell mix was transferred onto a CometSlide and placed in the dark at 4°C for 30 min. The slide containing the solidified gel was then placed in lysis solution at 4°C overnight. The next day, the slide was removed and then placed in alkaline unwinding solution for 20 min at room temperature. Pre-chilled alkaline electrophoresis solution was added to the CometAssay ES electrophoresis unit and the slide was placed into the chamber, where electrophoresis was performed at 18 V for 30 min. When complete, the slide was washed twice in water and once in 70% ethanol for 5 min each. The slide was dried at 37°C for 15 min and then stored at room temperature with a desiccant until ready for staining. The CometSlide was stained for 30 min using 100 *μ*l of SYBR-Green solution at room temperature. Afterwards, the CometSlide was dried and then imaged using a Zeiss Axio Observer Z1 inverted fluorescence microscope (Thornwood, NY, USA) with Hamamatsu ORCA-ER digital camera and AxioVision software. The values for 'Tail moment', a standard comet assay value that represents DNA damage and calculated as the product of the tail length and the fraction of total DNA in the tail (28,29), were then calculated from the images using CometScore software (Tritek Corporation, Sumerduck, VA, USA). A minimum of 200 cells for each treatment group were scored for quantification of tail moment.

### Statistical analyses

All error bars for calculated cell death and comet assay quantifications represent the standard error of the mean (SEM). Statistical analyses were accomplished by one-way analysis of variance (ANOVA) followed by the Tukey's and unpaired Student's t-tests. Statistical significance was defined as p<0.05.

## Results

### TRPM2 inhibition enhances cytotoxicity in MDA-MB-231 breast adenocarcinoma cells treated with chemotherapeutic agents

**Figure 1:**
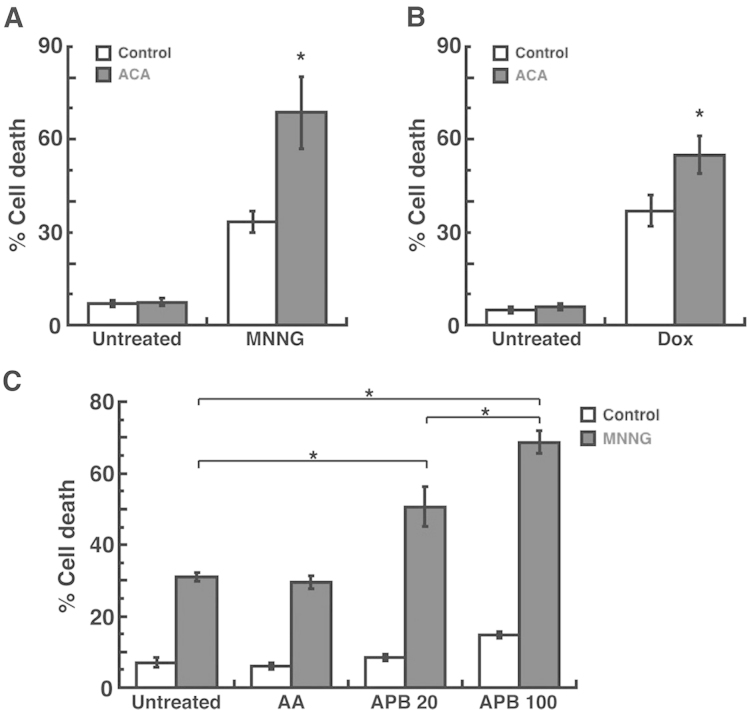
Cell death analyses of MDA-MB-231 breast adenocarcinoma cells after TRPM2 inhibition and chemotherapeutic treatments. (A) Quantification of cell death by flow cytometry in MDA-MB-231 breast adenocarcinoma cells. Cells were pretreated for 30 min with 20 *μ*M *N*-(*p*-amylcinnamoyl)anthranilic acid (ACA), a TRPM2 inhibitor, and subsequently treated with 100 *μ*M N-methyl-N′-nitro-N-nitrosoguanidine (MNNG) for 30 min. (B) Quantification of cell death by flow cytometry in MDA-MB-231 cells pretreated with 20 *μ*M ACA for 30 min and subsequently treated with 2 *μ*M doxorubicin (Dox) for 20 h. (C) Flow cytometric quantification of cell death in MDA-MB-231 cells pretreated with 100 *μ*M aristolochic acid (AA; a phospholipase A_2_ inhibitor), 20 *μ*M 2-aminoethoxydiphenyl borate (2-APB; a TRPM2 inhibitor) or 100 *μ*M 2-APB for 30 min and subsequently treated with 100 *μ*M MNNG for 30 min. All error bars represent the standard error of the mean (SEM). ^*^p<0.05 via one-way analysis-of-variance (ANOVA) and unpaired Student's t-test.

As we previously demonstrated that inhibition of TRPM2 led to decreased proliferation in breast adenocarcinoma cells (23), we next investigated the ability of TRPM2 inhibition to induce cell death. Treatment with the DNA methylating agent, N-methyl-N′-nitro-N-nitrosoguanidine (MNNG) (30), led to a significant level of cell death (35%) in the MDA-MB-231 breast adenocarcinoma cells (Fig. 1A). However, pretreatment with the TRPM2 inhibitor, *N*-(*p*-amylcinnamoyl) anthranilic acid (ACA) (31), enhanced the level of cell death to 68% in response to MNNG. Similar results were observed after treatment with doxorubicin, where the level of cell death caused by doxorubicin (38%) was significantly increased due to pretreatment with ACA (56%) (Fig. 1B). Since ACA was also reported to inhibit phospholipase A_2_ (32), we next utilized the phospholipase A_2_ inhibitor, aristolochic acid (33) in our studies. The results demonstrated that pretreatment of MDA-MB-231 breast adenocarcinoma cells with aristolochic acid, followed by treatment with MNNG, led to a cell death level of 29% (Fig. 1C). This level of cell death was not calculated to be significantly different than the level of cell death induced by MNNG alone (32%) (Fig. 1C). We also used an additional TRPM2 inhibitor, 2-aminoethoxydiphenyl borate (2-APB) (34), to further demonstrate the effects of TRPM2 inhibition on cell death in the MDA-MB-231 breast adenocarcinoma cells. The results demonstrated that pretreatment of these cells with 2-APB led to a dose-dependent increase in cell death after MNNG treatment, where 20 *μ*M 2-APB caused 48% cell death and 100 *μ*M caused 68% cell death in the MDA-MB-231 cells (Fig. 1C). Thus, the results of these studies indicated that inhibition of TRPM2 caused increased levels of cell death in the MDA-MB-231 breast adenocarcinoma cells after chemotherapeutic treatments. Moreover, this effect was most likely not mediated through phospholipase A_2_ inhibition. These results therefore demonstrated improved efficacy in the induction of cytotoxicity in breast cancer cells due to TRPM2 inhibition.

### TRPM2 RNAi silencing enhances cytotoxicity in triple-negative and estrogen receptor-positive breast adenocarcinoma cells treated with chemotherapeutic agents

**Figure 2:**
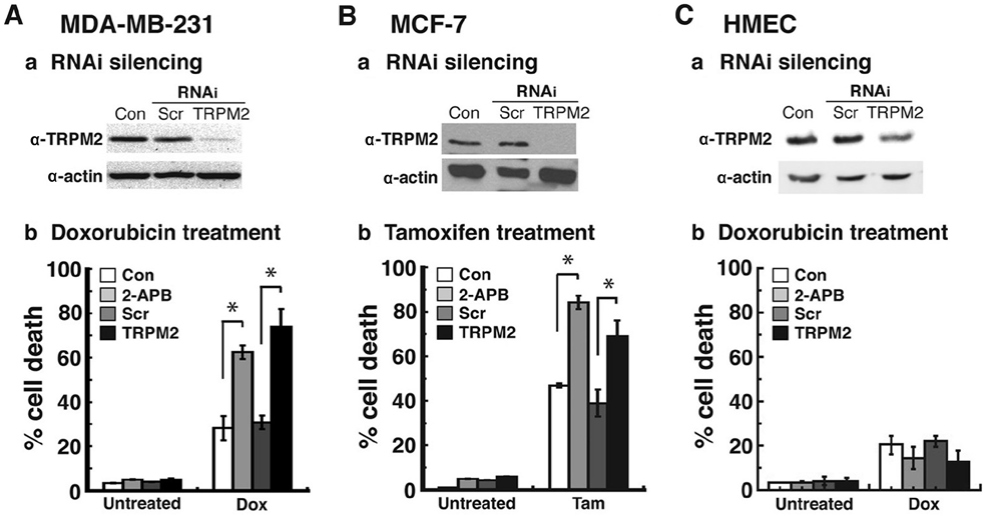
Effects of TRPM2 inhibition and RNAi knockdown in cancerous and non-cancerous breast cell lines after chemotherapeutic treatments. Immunoblot levels of TRPM2 protein after TRPM2 RNAi silencing (a) and quantification of cell death by flow cytometry (b) in (A) MDA-MB-231 breast adenocarcinoma, (B) MCF-7 breast adenocarcinoma and (C) human mammary epithelial cells (HMECs), a non-cancerous primary breast cell line. For RNAi silencing, cells were transfected with 100 nM anti-TRPM2 duplex siRNA oligos (TRPM2) or 100 nM negative control scrambled duplex siRNA oligos (Scr). Loading controls for immunoblots were provided by the immunodetection of β-actin. For chemotherapeutic treatments, the cells were pretreated 30 min with the TRPM2 inhibitor, 2-APB, and then subsequently treated with (A-b) 2 *μ*M doxorubicin (Dox) until analysis; (B-b) 7 *μ*M tamoxifen (Tam) until analysis; and (C-b) 2 *μ*M doxorubicin (Dox) until analysis. Con, untransfected negative control cells. All error bars represent the SEM. ^*^p<0.05 via one-way ANOVA and unpaired Student's t-test.

To demonstrate the specificity of chemotherapeutic effects due to decreased TRPM2 function, we utilized RNA interference to knock down the expression levels of TRPM2. We successfully decreased TRPM2 protein levels in the MDA-MB-231 breast adenocarcinoma cells by RNAi silencing (Fig. 2A–a). Treatment of this triple-negative (TN) breast cancer cell line with doxorubicin led to a 71% level of cell death (Fig. 2A–b). This level of cell death was similar to the level observed after 2-APB treatment in these cells (62%) (Fig. 2A–b). Furthermore, it was a significant increase from the level of cell death that resulted from doxorubicin treatment in the cells transfected with negative control scrambled (non-silencing) siRNA (30%).

We next investigated the effects of RNAi silencing of TRPM2 in the MCF-7 breast adenocarcinoma cell line, which is an estrogen receptor-positive breast cancer cell line (Fig. 2B). After successful RNAi silencing of TRPM2 in these cells (Fig. 2B–a), treatment with tamoxifen led to a 66% level of cell death, which was lower than the amount of cell death observed after 2-APB treatment (83%), yet significantly higher than the cell death exhibited by cells transfected with scrambled siRNA and treated with tamoxifen (36%) (Fig. 2B–b). Finally, in order to determine the effects of RNAi silencing in normal, non-cancerous breast cells, we successfully decreased TRPM2 protein levels in human mammary epithelial cells (HMECs) (Fig. 2C–a). Treatment of TRPM2-silenced HMECs with doxorubicin led to a 16% level of cell death, which was comparable to the 18% cell death observed in these cells after 2-APB treatment (Fig. 2C–b). These cell death levels were not significantly higher than the levels of cell death observed in the negative control groups (no pretreatment with a TRPM2 inhibitor or transfection with scrambled siRNA oligos). Moreover, the cell death levels in the TRPM2-silenced HMECs were significantly lower than the cell death levels in the TRPM2-silenced MDA-MB-231 and MCF-7 cells. Taken together, the results demonstrated that RNAi knockdown of TRPM2 levels caused increased levels of cell death in the MDA-MB-231 and MCF-7 cells after chemotherapeutic treatments. Furthermore, TRPM2 inhibition or RNAi silencing did not cause increased cell death in the non-cancerous HMECs. These studies therefore showed that decreased TRPM2 function led to enhanced cytotoxicity after chemotherapy in a TN breast cancer cell line and an ER+ breast cancer cell line, but not in a non-cancerous breast cell line.

### TRPM2 inhibition enhances DNA damage levels in MCF-7 breast adenocarcinoma cells treated with doxorubicin

**Figure 3:**
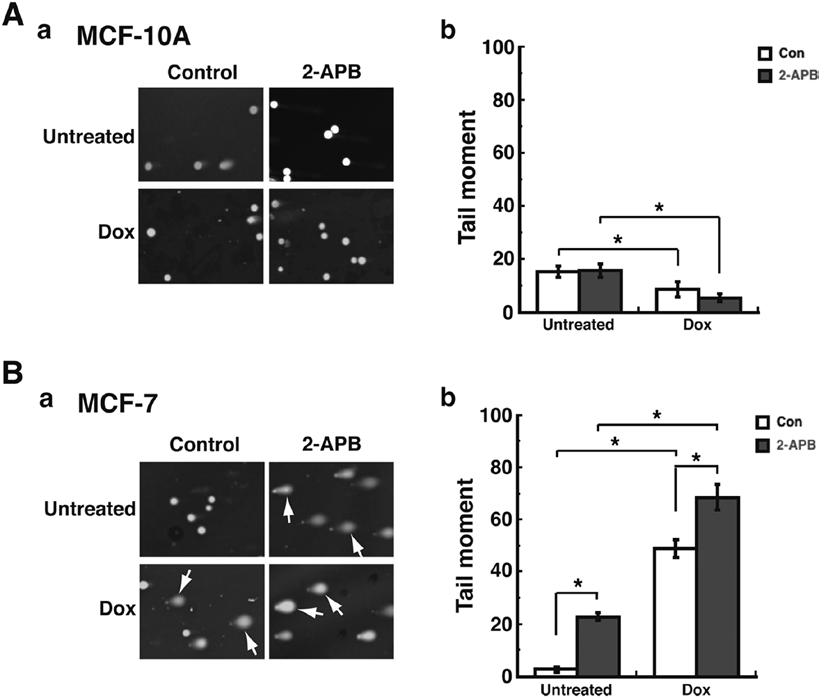
Analysis of DNA damage levels in cancerous and non-cancerous breast cell lines after TRPM2 inhibition and doxorubicin treatment. The non-cancerous MCF-10A human breast epithelial cell line (A) and the cancerous MCF-7 human breast adenocarcinoma cell line (B) were pretreated with 100 *μ*M 2-APB for 30 min, and then treated with 2 *μ*M doxorubicin (Dox). DNA damage levels were then analyzed by comet assay (a) and quantified by calculating the 'Tail moment' values (b), defined as the product of the tail length and fraction of the total DNA in the tail. Con, negative control cells not pretreated with 2-APB. ^*^p<0.05, one-way ANOVA and unpaired Student's t-test; error bars represent the SEM.

We previously demonstrated that TRPM2 inhibition or RNAi silencing caused increased levels of DNA damage in breast adenocarcinoma cells (23). We next investigated the potential ability of TRPM2 inhibition to increase the DNA damage levels induced by chemotherapy. To accomplish this, we utilized the single-cell gel electrophoresis (comet) assay. In the comet assay, cells are embedded in agarose on a microscope slide and then lysed to form nucleoids containing supercoiled DNA. Electrophoresis in alkaline conditions results in structures resembling comets, as observed by fluorescence microscopy. The intensity of the comet tail relative to the head corresponds to the amount of DNA strand breaks (35). Treatment of the non-cancerous MCF-10A cells with 2-APB with or without doxorubicin resulted in minimal levels of comets (Fig. 3A–a), which indicates minimal levels of DNA damage. This was quantified by calculating the 'Tail moment' values for each experimental group. Tail moment, a common quantification of comet assay results, is calculated as the product of the tail length and the fraction of total DNA in the tail (28,29). Thus, using CometScore software and analyzing a minimum of 200 cells/treatment group, the Tail moment values for the untreated MCF-10A cells and those pretreated with 2-APB were ~18 for each cohort (Fig. 3A–b). Treatment of these cells with doxorubicin led to decreased Tail moment values, which indicates decreased levels of DNA damage in MCF-10A cells. However, in the MCF-7 breast adenocarcinoma cells, increased levels of DNA damage were observed (Fig. 3B–b) after 2-APB pretreatment. This increased level of DNA damage was confirmed after quantification of Tail moment values, where the Tail moment value for untreated cells was 3, while the value for 2-APB pretreatment was 22 (Fig. 3B–b). Furthermore, while treatment with doxorubicin caused increased levels of DNA damage in these cells, pretreatment with 2-APB enhanced the level of DNA damage observed after doxorubicin treatment (Fig. 3B–a). This was also quantified, as the Tail moment value for doxorubicin treatment was 44, while the value for doxorubicin + 2-APB was 65 (Fig. 3B–b). Moreover, these enhanced levels of DNA damage due to 2-APB and doxorubicin treatment were statistically significant when compared to the DNA damage levels due to doxorubicin alone or 2-APB alone (Fig. 3B–b). These results thus indicated that the inhibition of TRPM2 caused increased levels of DNA damage in the MCF-7 breast adenocarcinoma cells after chemotherapeutic treatment. Furthermore, the results also demonstrated that TRPM2 inhibition did not lead to increased DNA damage levels in the non-cancerous breast cells.

### Increased cell death in MDA-MB-231 breast adenocarcinoma cells due to TRPM2 inhibition is not primarily mediated via apoptosis or autophagy

**Figure 4:**
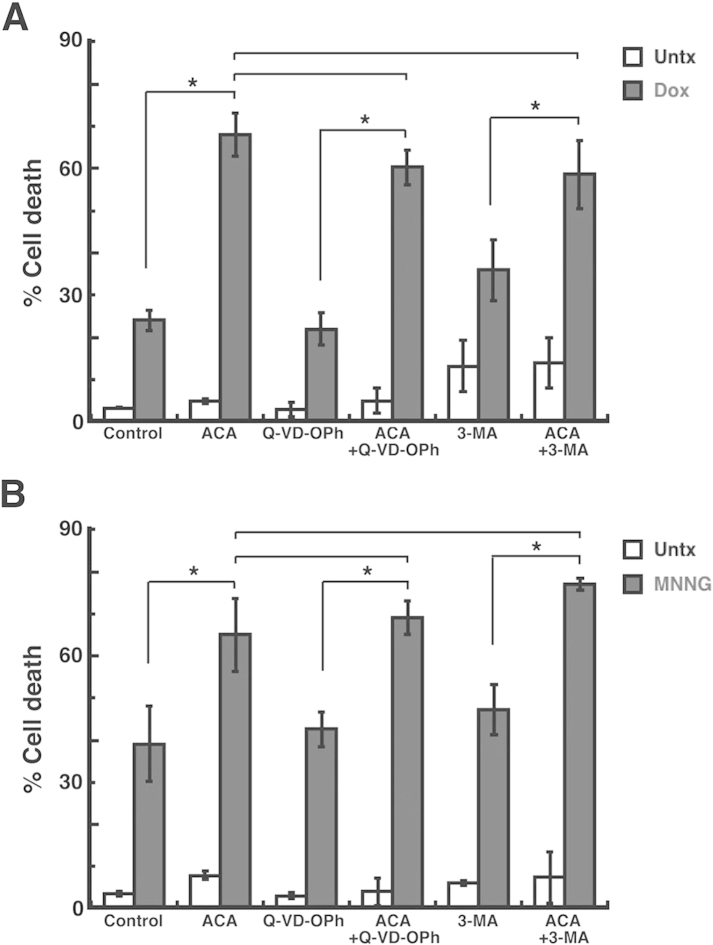
Analysis of apoptosis and autophagy in breast adenocarcinoma cells after TRPM2 inhibition and chemotherapeutic treatments. (A) MDA-MB-231 breast adenocarcinoma cells were pretreated with 20 *μ*M ACA for 30 min, and then treated with 2 *μ*M doxorubicin (Dox). Cell death was then quantified by flow cytometry. For analysis of apoptosis, cells were pretreated with 50 *μ*M Q-VD-OPh, a pan-caspase inhibitor. For analysis of autophagy, cells were pretreated with 3-methyladenine (3-MA), an inhibitor of autophagy. (B) MDA-MB-231 breast adenocarcinoma cells were treated as in A, except a 30-min exposure to 100 *μ*M MNNG was used as the chemotherapeutic treatment. ^*^p<0.05, one-way ANOVA and unpaired Student's t-test; error bars represent the SEM.

Since the aforementioned studies demonstrated increased cell death in breast adenocarcinoma cells after TRPM2 inhibition, we next investigated the potential cell death pathways induced after TRPM2 inhibition. To investigate apoptosis, we utilized Q-VD-OPh, an inhibitor of apoptosis due to its ability to function as a pan-caspase inhibitor (36). Pretreatment of the MDA-MB-231 breast adenocarcinoma cells with Q-VD-OPh led to a cell death level of 59% after ACA and doxorubicin treatment (Fig. 4A). However, this level of cell death was not significantly decreased when compared to the cell death induced by ACA and doxorubicin without Q-VD-OPh pretreatment (63%) (Fig. 4A). To investigate autophagy, we utilized the autophagy inhibitor, 3-methyladenine (3-MA) (37). Pretreatment with 3-MA led to 57% cell death in the MDA-MB-231 breast adenocarcinoma cells after ACA and doxorubicin treatment (Fig. 4A). This level of cell death was also not significantly different from the amount of cell death induced by ACA and doxorubicin without 3-MA pretreatment (Fig. 4A). Similar results were observed when the chemotherapeutic agent utilized was MNNG. Q-VD-OPh pretreatment led to 65% cell death after ACA and MNNG treatment, which was not significantly different than the amount of cell death caused by ACA + MNNG alone (63%) (Fig. 4B). Pretreatment with 3-MA led to 77% cell death in the MDA-MB-231 cells after ACA + MNNG. However, when compared to the cell death induced by ACA + MNNG (63%), this difference in cell death was not found to be statistically significant. The results thus demonstrated that – and 3-MA both failed to significantly decrease the amount of cell death observed after TRPM2 inhibition and chemotherapeutic treatments. Therefore, the studies suggest that apoptosis and autophagy are not the primary pathways of cell death induced by TRPM2 inhibition in breast adenocarcinoma cells after chemotherapy.

### Increased cell death in MDA-MB-231 breast adenocarcinoma cells due to TRPM2 inhibition is not primarily due to caspase-independent cell death mediated by poly(ADP-ribose) and apoptosis-inducing factor

**Figure 5:**
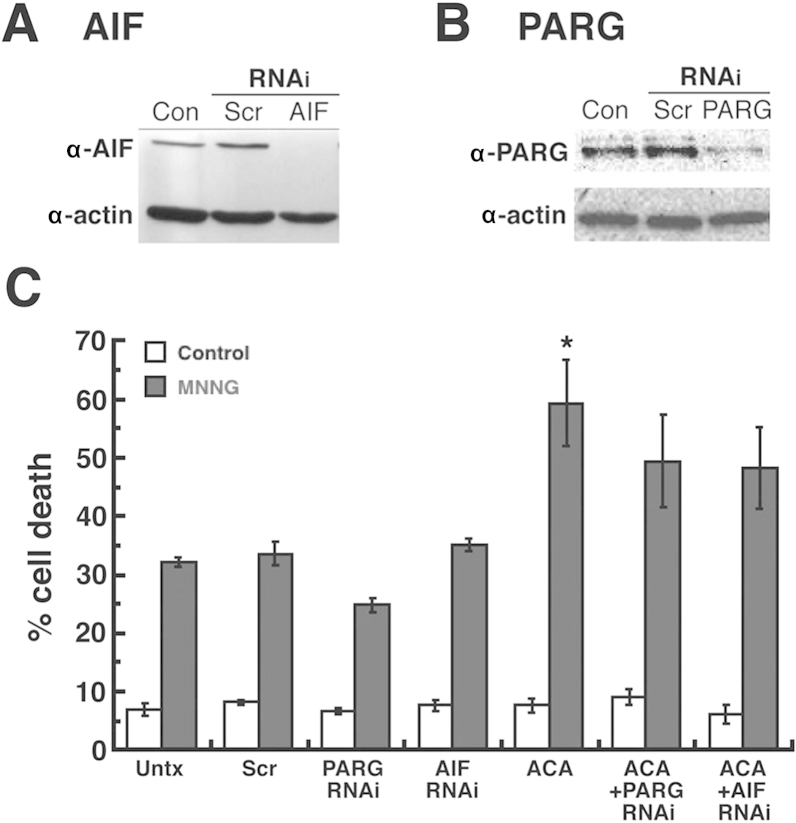
Analysis of poly(ADP-ribose)-mediated caspase-independent cell death in breast adenocarcinoma cells after TRPM2 inhibition and chemotherapeutic treatments. Immunoblot detection of (A) apoptosis-inducing factor (AIF) and (B) poly(ADP-ribose) glycohydrolase (PARG) in MDA-MB-231 breast adenocarcinoma cells after RNAi silencing. Loading controls for immunoblots were provided by the immunodetection of β-actin. Con, untransfected cells; Scr, cells transfected with negative control scrambled siRNA oligos. (C) Quantification of cell death by flow cytometry was performed in MDA-MB-231 cells after RNAi knockdown of AIF or PARG, pretreatment with 20 *μ*M ACA for 30 min and treatment with 100 *μ*M MNNG. ^*^p<0.05, one-way ANOVA and unpaired Student's t-test; error bars represent the SEM.

We continued our cell death analyses by investigating caspase-independent cell death. One caspase-independent pathway is cell death associated with poly(ADP-ribose) (PAR) and mediated by apoptosis-inducing factor (AIF) (38). PAR is an essential biopolymer to the cell that is synthesized by the PAR polymerases (PARPs) in response to DNA damage (39,40). High levels of PAR, due to high levels of DNA damage or the absence of PAR glycohydrolase (PARG) - the enzyme required to catabolize PAR - leads to caspase-independent cell death mediated by the pro-cell death protein, AIF (41,42). In addition, the activation and function of TRPM2 channels are known to be mediated by the metabolism of PA R (43). We thus utilized RNAi to knock down the expression of AIF and PARG in order to determine whether this pathway of caspase-independent cell death was a primary contributor to the increased cell death caused by TRPM2 inhibition. We successfully decreased AIF (Fig. 5A) and PARG (Fig. 5B) protein levels in the MDA-MB-231 breast adenocarcinoma cells by RNAi. Treatment of these cells with ACA and MNNG produced 50% cell death in the AIF-silenced cells and 49% cell death in the PARG-silenced cells (Fig. 5C). Both of these cell death values were lower than the cell death observed in the untransfected MDA-MB-231 cells treated with ACA + MNNG (59%), yet the difference was not found to be statistically significant. Thus, the results demonstrated that the RNAi knockdown of PARG and AIF expression did not significantly decrease the amount of cell death observed after TRPM2 inhibition and chemotherapeutic treatments. Therefore, the studies indicated that caspase-independent cell death mediated by PAR and AIF was not a primary pathway of cell death induced by TRPM2 inhibition in the breast adeno-carcinoma cells after chemotherapy.

## Discussion

We presented new data that further demonstrates the therapeutic potential of inhibiting TRPM2 function in the treatment of breast cancer. While we previously demonstrated a novel role for TRPM2 in breast adenocarcinoma cells, where it was shown to mediate DNA damage levels and cell proliferation, we expand upon these findings by reporting increased cell death due to inhibition of TRPM2. Furthermore, this was demonstrated in TN and ER^+^ breast cancer cell lines. Thus, the present study presents the possibility that targeting TRPM2 is expected to provide an additional strategy to successfully treat these molecular subtypes of breast cancer tumors. This is particularly important for TN breast cancer patients, as treatment options are limited, and the efficacy and prognosis of currently available treatments are not as favorable as those for patients with other molecular breast cancer subtypes. Therefore, the ability of TRPM2 inhibition to enhance cytotoxicity in TN and ER^+^ breast adenocarcinoma cells suggests that targeting TRPM2 may provide improved treatment options for TN and ER^+^ breast cancer patients in the future.

Our studies also provided important mechanistic and therapeutic insight into the cell death pathways induced by TRPM2 inhibition after chemotherapeutic treatments in breast adenocarcinoma cells. Increased cell death was observed in TN and ER^+^ breast adenocarcinoma cells after TRPM2 inhibition. This was corroborated with increased DNA damage levels in breast adenocarcinoma cells after chemotherapeutic treatments. Furthermore, the increased cell death observed was not found to be primarily mediated by apoptotic, autophagic or caspase-independent cell death pathways. It was particularly unexpected that caspase-independent cell death induced by PAR and mediated by AIF was not a primary pathway that was triggered by TRPM2 inhibition. This is because TRPM2 function is regulated by PAR metabolism, where ADP-ribose moieties generated due to the catabolism of PAR polymers by PARG have been shown to activate the gating of cations by TRPM2 channels. Due to this regulation of TRPM2 channels by PAR, along with the ability of activated TRPM2 channels to facilitate cell death in non-cancerous cells, the lack of caspase-independent cell death after TRPM2 inhibition in breast adenocarcinoma cells was unexpected. However, our studies demonstrated that TRPM2 appears to have a novel role in breast cancer cells, while Zeng *et al* previously demonstrated a potentially novel role for TRPM2 in prostate cancer cells (22). Furthermore, our observation of the lack of PAR-mediated cell death in breast cancer cells after TRPM2 inhibition, along with the observation by Zeng *et al* of the failure of PAR to mediate TRPM2 function in prostate cancer cells, appears to corroborate this novel role in both breast and prostate cancer cells. Thus, it is conceivable that the novel role for TRPM2 in cancer cells is the basis for the observation that inhibition of TRPM2 produces novel chemotherapeutic effects in cancer cells, with minimal deleterious effects in non-cancerous cells.

Additional therapeutic insight gained from these results is that TRPM2 inhibition has the potential to eradicate breast cancer cells that are resistant to chemotherapy due to their evasion of apoptosis. Our preliminary findings indicate that TRPM2 inhibition is expected to induce alternative cell death pathways in breast adenocarcinoma cells. It is therefore possible that TRPM2 inhibition could provide the same effects in breast cancer cells that are refractive to chemotherapy, particularly those that evade apoptotic cell death, and thus survive after chemotherapy. This is a significant finding, since breast tumors that are not responsive to chemotherapy are a cause for significant morbidity and mortality in breast cancer patients. The ability to overcome this resistance to chemotherapy would clearly lead to improvements in breast cancer chemotherapeutic treatments, and the overall survival and prognosis of breast cancer patients in the future. Thus, our results offer the possibility that targeting TRPM2 in breast tumors refractive to chemotherapeutic treatments may lead to the improved eradication of such tumors.

Future studies will be required to identify the primary cell death pathway(s) induced by TRPM2 inhibition. The lack of a primary role for apoptosis, autophagy or PAR-mediated caspase-independent cell death in breast adenocarcinoma cells after TRPM2 inhibition and chemotherapeutic treatments suggests that necrosis is the primary cell death pathway induced. This is a viable possibility, as a previous study demonstrated the exacerbation of necrotic cell death due to TRPM2 activation (24). However, this study was accomplished in non-cancerous cells. Furthermore, the clinical significance of other potential alternative cell death pathways are beginning to emerge. For example, TRPM2 inhibition in cardiac and neuroblastoma cells resulted in the upregulation of mitophagy (21,44). Thus, more studies are required in order to determine the primary cell death pathway(s) involved in breast adenocarcinoma cells after TRPM2 inhibition.

Future studies will also be required to characterize and identify the cellular effects of TRPM2 in breast cancer cells. These mechanistic studies will be particularly important in order to determine whether TRPM2 has different roles, not only in cancerous vs. non-cancerous cells, but also among different types of cancers. Current data are suggestive, yet not conclusive, that TRPM2 may indeed have different roles in various types of cancers. Our previous study in breast cancer cells, along with the study by Zeng *et al* that investigated TRPM2 in prostate cancer cells, determined that TRPM2 has a nuclear localization in breast and prostate cancer cells. This localization was in contrast to the currently known localization of TRPM2, where it functions as an ion channel in the plasma membrane and lysosomal membrane. However, in a well-designed recent report, the differential role of TRPM2 was found to be dependent on the activities of TRPM2 isoforms, where a truncated TRPM2 isoform (TRPM2-S) was found to decrease levels of the transcription factors, HIF-1 and HIF-2 (21). This led to cytotoxic effects in neuroblastoma cells. However, these studies demonstrated a localization of TRPM2 in the plasma and lysosomal membranes. Based on the studies to date in three different types of cancer, it is possible that TRPM2 may have different localizations and different roles in various types of cancer. Future studies will be required to determine whether TRPM2 does indeed have novel roles in different types of cancer.

In conclusion, we demonstrated that TRPM2 inhibition leads to increased cell death, increased DNA damage, and the induction of alternative pathways of cell death. This enhanced and novel induction of alternative cell death appears to be possible in TN and ER^+^ breast cancer cells. It also has the potential to lead to the targeting and successful eradication of chemotherapy-resistant breast cancer cells in the future. Thus, the present study provides compelling evidence that TRPM2 is a novel target that can lead to improved outcomes in the treatment of breast cancer patients in the future.

## Abbreviations

2-APB: 2-aminoethoxydiphenyl borate
3-MA: 3-methyladenine
AA: aristolochic acid
ACA: N-(p-amylcinnamoyl) anthranilic acid
AIF: apoptosis-inducing factor
ER+: estrogen receptor-positive
MNNG: N-methyl-N'-nitro-N-nitrosoguanidine
PAR: polyadenosine diphosphoribose biopolymer
PARG: poly-(ADP-ribose) glycohydrolase
TN: triple-negative
TRP: transient receptor potential superfamily of cation channels
TRPM: transient receptor potential, melastatin subfamily
